# Proficiency, Clarity, and Objectivity of Large Language Models Versus Specialists’ Knowledge on COVID-19's Impacts in Pregnancy: Cross-Sectional Pilot Study

**DOI:** 10.2196/56126

**Published:** 2025-02-05

**Authors:** Nicola Luigi Bragazzi, Michèle Buchinger, Hisham Atwan, Ruba Tuma, Francesco Chirico, Lukasz Szarpak, Raymond Farah, Rola Khamisy-Farah

**Affiliations:** 1 Laboratory for Industrial and Applied Mathematics Department of Mathematics and Statistics York University Toronto, ON Canada; 2 Department of Computer Science, Data Science, and Information Technology, Faculty of Natural and Applied Sciences, Sol Plaatje University Kimberley South Africa; 3 Azrieli Faculty of Medicine Bar-Ilan University Ramat Gan Israel; 4 Kaplan Medical Centre Department of Internal Medicine Hebrew University Rehovot Israel; 5 Department of Obstetrics and Gynecology Galilee Medical Center Nahariya Israel; 6 Post-Graduate School of Occupational Health Università Cattolica del Sacro Cuore Rome Italy; 7 Henry JN Taub Department of Emergency Medicine Baylor College of Medicine Houston, TX United States; 8 Department of Clinical Research and Development, LUXMED Group Warsaw Poland; 9 Institute of Medical Science, Collegium Medicum, The John Paul II Catholic University of Lublin Lublin Poland

**Keywords:** COVID-19, vaccine, reproductive health, generative artificial intelligence, large language model, chatGPT, google bard, microsoft copilot, vaccination, natural language processing, obstetric, gynecology, women, text mining, sentiment, accuracy, zero shot, pregnancy, readability, infectious

## Abstract

**Background:**

The COVID-19 pandemic has significantly strained health care systems globally, leading to an overwhelming influx of patients and exacerbating resource limitations. Concurrently, an “infodemic” of misinformation, particularly prevalent in women’s health, has emerged. This challenge has been pivotal for health care providers, especially gynecologists and obstetricians, in managing pregnant women’s health. The pandemic heightened risks for pregnant women from COVID-19, necessitating balanced advice from specialists on vaccine safety versus known risks. In addition, the advent of generative artificial intelligence (AI), such as large language models (LLMs), offers promising support in health care. However, they necessitate rigorous testing.

**Objective:**

This study aimed to assess LLMs’ proficiency, clarity, and objectivity regarding COVID-19’s impacts on pregnancy.

**Methods:**

This study evaluates 4 major AI prototypes (ChatGPT-3.5, ChatGPT-4, Microsoft Copilot, and Google Bard) using zero-shot prompts in a questionnaire validated among 159 Israeli gynecologists and obstetricians. The questionnaire assesses proficiency in providing accurate information on COVID-19 in relation to pregnancy. Text-mining, sentiment analysis, and readability (Flesch-Kincaid grade level and Flesch Reading Ease Score) were also conducted.

**Results:**

In terms of LLMs’ knowledge, ChatGPT-4 and Microsoft Copilot each scored 97% (32/33), Google Bard 94% (31/33), and ChatGPT-3.5 82% (27/33). ChatGPT-4 incorrectly stated an increased risk of miscarriage due to COVID-19. Google Bard and Microsoft Copilot had minor inaccuracies concerning COVID-19 transmission and complications. In the sentiment analysis, Microsoft Copilot achieved the least negative score (–4), followed by ChatGPT-4 (–6) and Google Bard (–7), while ChatGPT-3.5 obtained the most negative score (–12). Finally, concerning the readability analysis, Flesch-Kincaid Grade Level and Flesch Reading Ease Score showed that Microsoft Copilot was the most accessible at 9.9 and 49, followed by ChatGPT-4 at 12.4 and 37.1, while ChatGPT-3.5 (12.9 and 35.6) and Google Bard (12.9 and 35.8) generated particularly complex responses.

**Conclusions:**

The study highlights varying knowledge levels of LLMs in relation to COVID-19 and pregnancy. ChatGPT-3.5 showed the least knowledge and alignment with scientific evidence. Readability and complexity analyses suggest that each AI’s approach was tailored to specific audiences, with ChatGPT versions being more suitable for specialized readers and Microsoft Copilot for the general public. Sentiment analysis revealed notable variations in the way LLMs communicated critical information, underscoring the essential role of neutral and objective health care communication in ensuring that pregnant women, particularly vulnerable during the COVID-19 pandemic, receive accurate and reassuring guidance. Overall, ChatGPT-4, Microsoft Copilot, and Google Bard generally provided accurate, updated information on COVID-19 and vaccines in maternal and fetal health, aligning with health guidelines. The study demonstrated the potential role of AI in supplementing health care knowledge, with a need for continuous updating and verification of AI knowledge bases. The choice of AI tool should consider the target audience and required information detail level.

## Introduction

The COVID-19 pandemic has exerted unprecedented strain on health care systems worldwide, overwhelming hospitals and health care facilities with patients and stretching resources and staff to their limits, thus highlighting the critical need for efficient health care delivery, and underscoring the importance of robust, resilient public health infrastructure [1].

In addition, the pandemic gave rise to an “infodemic”—that is to say, a flood of information, often misleading or false, that spread through social media and other channels [2], which has significantly impacted maternal and child health [3]. This deluge of misinformation made the task of retrieving and providing reliable, science-based information more challenging yet crucial [4] and health care providers, including gynecologists and obstetricians, played a pivotal role during the pandemic, especially in advising pregnant women.

Pregnancy, by its nature, puts women at a higher risk for complications from viral infections like COVID-19 [5,6]. As vaccines became available, gynecologists and obstetricians were at the forefront, recommending COVID-19 vaccinations to pregnant women, and having to balance the lack of initial data on vaccine safety in pregnancy with the known risks of COVID-19, providing tailored advice to their patients. The role of these specialists, crucial in safeguarding the health of mothers and their unborn children, faced significant challenges during the pandemic. This period adversely affected their professional relationships with patients and colleagues, leading to anxiety, frustration, and physical strain. These stressors, stemming from both the workplace and pandemic conditions, have prompted some gynecologists and obstetricians to consider early retirement or leaving the profession, highlighting an increased risk of burnout among these specialists [7].

The advent of generative artificial intelligence (AI), such as large language models (LLMs) and related disruptive technologies, has shown immense potential in various fields, including health care [8]. These AI-based systems hold great potential in assisting and supporting biomedical researchers, health care professionals, and the general public. They can process vast amounts of data, provide medical insights, assist in diagnostic processes, and even support therapies. In the context of the COVID-19 pandemic, AI has been used not only to analyze virus structures, predict outbreaks, and sift through vast amounts of research to identify potential treatments [9] but also to respond to queries from people, providing accurate and timely information on symptoms, prevention measures, and vaccination.

However, with the rapid development and deployment of these AI-driven technologies, there arises an essential need to test the knowledge and proficiency of AI-based systems rigorously [10]. Ensuring the accuracy, reliability, and ethical use of AI is vital, especially in sensitive areas like health care, and involves continuous monitoring, evaluation, and updating of AI systems to align with the latest scientific knowledge and ethical standards. As AI becomes more integrated into human lives, the necessity for such oversight becomes increasingly important to prevent misuse and ensure that these powerful tools serve the greater good effectively and responsibly.

Recent studies have explored the potential use of AI and LLMs in public health initiatives, particularly in the context of immunization campaigns [11-20]. These investigations have demonstrated that AI can play a critical role in countering misinformation and dismissing conspiracy theories, thereby fostering trust and acceptance of vaccines among the public. By providing clear, evidence-based responses to common myths and misconceptions, AI-driven systems can effectively address vaccine hesitancy and promote informed decision-making. However, besides being limited in number, these studies are also constrained in scope, often concentrating on individual models without providing a comprehensive comparative analysis of their performance. This lack of comparative insight leaves gaps in understanding the relative strengths and weaknesses of different AI systems in supporting public health efforts, highlighting the need for broader investigations that include a variety of generative AI tools to fully assess their potential and limitations in these critical domains.

As of today, the landscape of AI prototypes is dominated by 3 leading models, that are ChatGPT-4 (which has surpassed its predecessor, ChatGPT-3.5), Microsoft Copilot, and Google Bard (now rebranded as Google Gemini). ChatGPT-3.5, released by OpenAI and based on the GPT-3.5 model, is a conversational AI model designed primarily for text-based interactions, and skilled in a variety of tasks like answering questions, providing explanations, and generating text based on prompts, among others. The model’s knowledge is up to the date it was last trained, which means it does not have information on events or developments that occurred after that date. ChatGPT-4 represents its further iteration. As such, this advanced version by OpenAI, succeeding ChatGPT-3.5, built on the GPT-4 architecture, offers enhanced capabilities in terms of understanding and generating more complex and nuanced text, and is more contextually aware, providing more relevant and accurate responses. Like its predecessor, it is trained up to a certain point in time and lacks real-time knowledge. Microsoft Copilot is primarily focused on assisting with coding, problem-solving, and programming tasks and can be integrated with development tools like GitHub and Visual Studio Code (Microsoft Inc). Finally, Google Bard, powered by Google’s own language models, including BERT, is designed to integrate Google’s search capabilities, potentially offering more up-to-date information, and is aimed at providing conversational AI experiences, including answering questions or generating text, with a strong emphasis on leveraging the web for current information.

Each of these models has unique strengths and applications, with ChatGPT models (3.5 and 4) focusing on general conversational tasks, Microsoft Copilot on programming assistance, and Google Bard on integrating search capabilities into conversational AI. As such, each of these LLMs was tested in the present investigation. More in detail, the research objective of this pilot study was to evaluate the applicability of major generative AI model prototypes in providing evidence-based responses to COVID-19–related queries, particularly in the context of maternal and child health. The evaluation focused on key aspects such as accuracy, reliability, and readability. By benchmarking these models against a range of factual statements, the study aimed to assess their potential not only to support health care professionals in decision-making and public health communication during pandemics but also to address the challenges faced by the public in accessing accurate and reliable information.

The findings will help determine the role of AI in enhancing health care delivery and improving public understanding of critical health issues during crises. By highlighting the dual challenge of supporting specialists and ensuring the public is correctly informed, this research underscores the complex interplay between information dissemination and health care communication. As a pilot study, it serves as a foundational step, emphasizing the need for further, more comprehensive investigations to fully explore and validate the capabilities and limitations of AI in health care and public health contexts.

## Methods

### Procedure

All 4 AI-based tools were queried about COVID-19 and related vaccines during pregnancy using zero-shot prompts, that is to say, without any previous examples or context specific to that task. They were queried all on the same day, on January 7, 2024.

In a comparative evaluation of ChatGPT-3.5, ChatGPT-4, Google Bard, and Microsoft Copilot, these AI-based tools were tested using a validated questionnaire. This had previously been administered to a sample of 159 Israeli obstetrician-gynecologists, with an average age of 44.9 years (SD 12.48), predominantly female (59.7%), and largely composed of attending physicians (60.4%) [21]. This assessment aimed to evaluate the AIs’ ability to provide accurate information on COVID-19, particularly in the context of pregnancy, fertility, and related clinical issues, as well as to make evidence-based recommendations. The questionnaire was developed following a comprehensive review of the literature and in accordance with World Health Organization (WHO) guidelines. It consisted of 5 sections designed to capture a range of relevant information. The first section collected sociodemographic data, including participants’ gender, age, marital status, number of children, religion, level of religious observance, professional role (resident or attending physician), years of practice, as well as previous COVID-19 infection and vaccination status. The second section assessed general knowledge about COVID-19 through 5 true or false questions that addressed basic preventive measures, transmission routes, and management strategies. The third section focused on specific knowledge related to pregnancy, childbirth, and breastfeeding during the COVID-19 pandemic, including 14 questions exploring the risks and complications of COVID-19 for pregnant women, the potential for vertical transmission, the safety of breastfeeding, and related clinical practices. The fourth section addressed knowledge of COVID-19 vaccination through 14 questions that covered vaccine types, safety, efficacy, administration during pregnancy, and potential side effects. Finally, the fifth section examined attitudes and practices regarding COVID-19 vaccination. This section explored physicians’ recommendations about vaccination timing during pregnancy, vaccination for women planning pregnancy, and associated safety considerations, with responses recorded on a 5-point Likert scale. To ensure clarity and relevance, the questionnaire was piloted with 12 obstetrician-gynecologists before full deployment. The survey was then distributed using both digital platforms and in-person delivery during professional gatherings, achieving a high response rate. Logistic regression analysis of the data revealed that knowledge of COVID-19 vaccination was a significant predictor of whether obstetrician-gynecologists recommended vaccination for pregnant women.

In the present comparative assessment, items from the second through fifth sections of the questionnaire were used. Sections 2 to 4 were included in their original format to specifically evaluate the AI systems’ proficiency in delivering accurate knowledge about COVID-19 prevention, risks during pregnancy, and vaccination safety and efficacy. For section 5, instead of using the original Likert scale, the AI tools were queried with direct recommendation prompts such as “Would you recommend...?” The responses from section 5 were subsequently analyzed for readability and sentiment (refer to next sections), providing insights into the clarity and tone of the AI-generated recommendations.

All the queried items, along with the corresponding prompts, are detailed in Multimedia Appendix 1.

### Scoring

The scoring process of the replies provided by the four AI models (reported in Multimedia Appendix 2) was conducted independently by 2 obstetrician-gynecologists (RT and RK-F) and 1 internal medicine doctor (RF). To ensure impartiality, the biostatistician (NLB) analyzing the data was blinded to the identities of the LLMs during the scoring process.

### Sentiment Analysis

Textual data analysis of AI-generated responses to the items of the fifth section of the validated questionnaire (Multimedia Appendix 3) was carried out using XLSTAT (Data Analysis and Statistical Solution for Microsoft Excel, Addinsoft) for each LLM-generated text to ensure comprehensive and model-specific insights. Each text underwent preprocessing to prepare it for analysis. This included removing English stop words, punctuation, and numerical characters, as well as filtering out words with a frequency of less than two occurrences. A sparsity threshold of 0.975 was applied to exclude terms appearing in fewer than 2.5% of the dataset, resulting in a more refined and representative corpus. Following preprocessing, a Term-Document Matrix (TDM) was constructed, providing a structured representation of the frequency of each term across the documents generated by the LLMs. The TDM facilitated comparative and statistical analyses by organizing the data into a matrix format, with columns representing unique terms and rows labeling the specific LLM. A Bag-of-Words representation was derived to enable both word frequency visualization and sentiment evaluation. Word clouds were generated to visually represent the most frequently occurring words for each LLM-generated text. The size and prominence of the words in the cloud reflected their frequency, offering an intuitive summary of the dominant themes and linguistic patterns specific to each model. To assess the emotional tone and polarity of the texts, sentiment analysis was performed using the TDM. Tokenization was applied to isolate individual words, and the Bing sentiment lexicon was used to assign binary polarity scores (positive or negative) to each term. These scores were then aggregated to calculate the overall sentiment for each LLM-generated text.

### Readability Analysis

The readability of the 4 AI’s responses to the items of the fifth section of the validated questionnaire (Multimedia Appendix 3) was appraised by means of the Flesch-Kincaid Grade Level analysis, which is a test designed to indicate how difficult a passage in English is to understand. The formula was developed by Rudolf Flesch and J Peter Kincaid and was first used in the United States military to assess the readability of their technical manuals. It is widely used in the field of education, but also in other sectors that require clarity of written communication, such as legal and health care industries. The formula is based on 2 factors, that are the average number of words per sentence and the average number of syllables per word. The formula is, Flesch-Kincaid Grade Level=0.39 × (average number of words per sentence) + 11.8 × (average number of syllables per word) − 15.59

The result of the Flesch-Kincaid Grade Level formula is a number that corresponds with a US school grade level. For example, a score of 8 means that the document can be understood by an eighth grader, with a higher score indicating more complex and difficult text.

In addition, the Flesch Reading Ease Score was also used to evaluate the readability of the responses. This score rates text on a 100-point scale, where higher scores indicate easier-to-read material. The formula for calculating the Flesch Reading Ease Score is Flesch Reading Ease Score = 206.835 − 1.015 × (average number of words per sentence) − 84.6 × (average number of syllables per word)

Higher scores on this scale indicate easier-to-read text, while lower scores indicate more complex material. For example, scores above 90 suggest very easy text suitable for an average 10- to 11-year-old (5th grade), while scores below 30 indicate very difficult text meant for college graduates.

### Ethical Considerations

The primary study [21], which provided data from human participants, was exempted from formal approval by the Ethics Committee of the Medical Faculty at Bar-Ilan University in Tel Aviv, Israel, due to its survey-based design. Participants, consisting of obstetrician-gynecologists (both residents and attending physicians), voluntarily consented to participate in the study without receiving any compensation. All data were fully anonymized, ensuring the privacy and confidentiality of the participants. In addition, no ethical approval was required for the AI component of this study.

## Results

### LLMs’ Knowledge of COVID-19 and Its Vaccines for Maternal and Fetal Health

ChatGPT-4 and Microsoft Copilot obtained a score of 97% (32/33), while Google Bard scored 94% (31/33), and ChatGPT-3.5 scored 82% (27/33; Multimedia Appendix 3).

ChatGPT-4 delivered correct responses across a range of topics, demonstrating a high level of proficiency in this specialized field. However, there was a notable exception in its performance: ChatGPT incorrectly stated that there is a heightened risk of miscarriage among pregnant women with COVID-19, even though, in reality, according to current literature, there is no evidence to support an increased risk of miscarriage due to COVID-19.

Similarly, Microsoft Copilot made only one mistake, related to the item concerning the increased risk of complications for pregnant women with COVID-19 compared to nonpregnant women of the same age. Google Bard’s knowledge of COVID-19 and its vaccines for women’s health was rather good, with the AI-based tool making only 2 mistakes, by ruling out COVID-19 intrauterine transmission, transmission during delivery, or through contact and respiratory droplets during breastfeeding.

On the contrary, ChatGPT-3.5 exhibited poorer performance, with 6 wrong responses, demonstrating gaps in the knowledge of (1) risk, (2) complications of COVID-19 in pregnant women, (3) medication for pregnant and postpartum women with COVID-19, (4) risk of miscarriage in COVID-19–infected pregnant women, (5) mother-to-child transmission of COVID-19, and (6) COVID-19 transmission through breast milk.

### LLMs’ Attitudes and Practices Toward COVID-19 and Its Vaccines for Maternal and Fetal Health

Even if ChatGPT-3.5, ChatGPT-4, Microsoft Copilot, and Google Bard, as AI-based LLMs, cannot and do not provide personal opinions or medical recommendations, they can offer up-to-date and reliable information based on current guidelines and scientific understanding regarding COVID-19 vaccinations. In particular, concerning the recommendation of COVID-19 vaccine to all pregnant women (without contraindication), ChatGPT-4 stated that many health organizations, including the Centers for Disease Control and Prevention and the WHO, generally recommend COVID-19 vaccination for pregnant women due to the increased risk of severe illness from COVID-19 during pregnancy (including hospitalization, intensive care unit admission, and death). Google Bard concurred, emphasizing that the benefits of COVID-19 immunization far outweigh any potential risks to the mother or baby.

In terms of the proper timing for recommending the immunization of pregnant women against COVID-19, the AI-based chatbot clearly stated that the recommendation for COVID-19 vaccination during pregnancy often applies to all trimesters and, especially in the case of doubts, the timing should be discussed with a health care provider. Google Bard added that there is no evidence that vaccination at any particular trimester poses a higher risk to the mother or baby and Copilot stated that emphasis should be on vaccine receipt as soon as possible to maximize maternal and fetal health.

When queried about the recommendation of the COVID-19 vaccine only to pregnant women at high risk of contracting the virus, both ChatGPT-4 and Google Bard posited that the current guidance from many health organizations is to offer the vaccine to all pregnant women, not just those at high risk, due to the potentially severe impacts of COVID-19 during pregnancy.

Concerning the recommendation of COVID-19 vaccine to all women (without contraindication) of reproductive age who are not pregnant, ChatGPT-4 advised that COVID-19 vaccination is widely recommended for women of reproductive age to prevent severe illness, complications, and potential long-term effects of COVID-19. For those planning to undergo assisted reproduction, according to both the 2 AI-based tools, they are generally advised to get vaccinated against COVID-19, as there is no evidence that vaccines can affect fertility. When queried about the recommended interval between vaccination and pregnancy, the OpenAI chatbot stated that there is no recommended interval between receiving a COVID-19 vaccine and becoming pregnant according to most health organizations. Google Bard emphasized that there is no clear consensus on whether there is a need for an interval between vaccination and pregnancy, with some experts believing that there may be a slight increase in the risk of miscarriage in the first few weeks after vaccination, but this risk is very small, and other experts believing that there is no need to wait for any period of time before trying to conceive after vaccination. According to Google Bard, more research is warranted to determine whether there is a need for an interval between vaccination and pregnancy.

Finally, when tasked with recommending an interval between COVID-19 vaccination and the use of assisted reproductive technology (ART), ChatGPT-4 clarified that there is no specified interval recommended between COVID-19 vaccination and undergoing ART, while, according to Google Bard, there is no need for an interval between vaccination and ART, in that COVID-19 vaccination can safely be administered to women undergoing ART at any time.

On the contrary, ChatGPT-3.5 failed to offer up-to-date and reliable information, recommending immunization against COVID-19 in pregnant women at high risk, especially during the third trimester as it would be safer than during the first and second trimesters. Furthermore, ChatGPT-3.5 recommended an interval between vaccination and pregnancy as well as between immunization and ART based on vaccine type and individual risk factors.

### LLMs’ Text-Mining Analysis

The comparative text-mining analysis of Google Bard, Microsoft Copilot, ChatGPT-3.5, and ChatGPT-4 revealed both similarities and differences in the vocabulary used when discussing COVID-19 and immunization in relation to pregnancy. While all the models highlighted core themes such as “pregnant,” “COVID-19,” “vaccination,” and “women,” the nuances in their focus and vocabulary indicated variations in their approach to presenting the information. Google Bard and Microsoft Copilot both emphasized terms related to pregnancy and COVID-19, but their focal points diverged. Google Bard included more detailed terms associated with vaccination recommendations and the effects of COVID-19 on pregnancy outcomes, suggesting an emphasis on clinical guidance. In contrast, Microsoft Copilot featured broader terminology, incorporating aspects like “risk” and “breastfeeding,” indicating a wider scope that touches on future planning and broader maternal and fetal health. When comparing Google Bard to ChatGPT-3.5 and ChatGPT-4, shared terms like “pregnant” and “vaccination” pointed to a shared understanding of key themes. However, Google Bard prioritized actionable guidance and recommendations, while ChatGPT variants included a more detailed vocabulary concerning medical and health-related risks. This distinction highlighted Google Bard’s focus on practical advice, compared to ChatGPT’s detailed explanations of risks and safety. Microsoft Copilot and the ChatGPT versions also shared several core terms but differed in their linguistic styles. Microsoft Copilot tended to lean toward broader generalizations, which makes it suitable for summarizing information succinctly. On the other hand, ChatGPT versions delved into specifics, incorporating medical and technical language to provide more comprehensive information about COVID-19 and its impact on pregnancy. This distinction reflected the contrast between Copilot’s generalist approach and ChatGPT’s detailed focus. The comparison between ChatGPT-3.5 and ChatGPT-4 revealed a significant overlap in vocabulary, indicating consistency between the 2 versions. However, ChatGPT-4 introduced additional terms and nuances, suggesting an evolution in depth and breadth of content. These differences highlighted improvements in ChatGPT-4’s ability to provide detailed and nuanced medical insights compared to its predecessor.

### LLMs’ Readability Analysis

The Flesch-Kincaid Grade Level and Flesch Reading Ease Score for Google Bard’s response were 12.9 and 35.8, respectively, indicating that the text was highly complex and best understood by individuals with postsecondary education. Microsoft Copilot scored 9.9 and 49, suggesting a less complex response that was easier to read. Among the ChatGPT models, ChatGPT-3.5 exhibited a similar level of complexity to Google Bard, with scores of 12.9 and 35.6. ChatGPT-4, with scores of 12.4 and 37.1, was slightly less complex than both ChatGPT-3.5 and Google Bard but more complex than Microsoft Copilot. Overall, ChatGPT responses were best suited for readers with advanced reading skills, with ChatGPT-3.5 requiring graduate-level comprehension and ChatGPT-4 being more appropriate for advanced undergraduate-level readers (Table 1).

**Table 1:** Readability analysis for each large language model tested (Google Bard, Microsoft Copilot, ChatGPT-3.5, and ChatGPT-4).

AI Model	Flesch-Kincaid Grade Level	Flesch Reading Ease Score	Reading Complexity	Target Audience
Google Bard	12.9	35.8	Highly complex	Post-secondary education
Microsoft Copilot	9.9	49	Less complex	High school to early undergraduate level
ChatGPT-3.5	12.9	35.6	Similar complexity to Google Bard	Graduate-level comprehension
ChatGPT-4	12.4	37.1	Slightly less complex than ChatGPT-3.5 and Bard	Advanced undergraduate-level comprehension

### LLMs’ Sentiment Analysis

**Figure 1:**
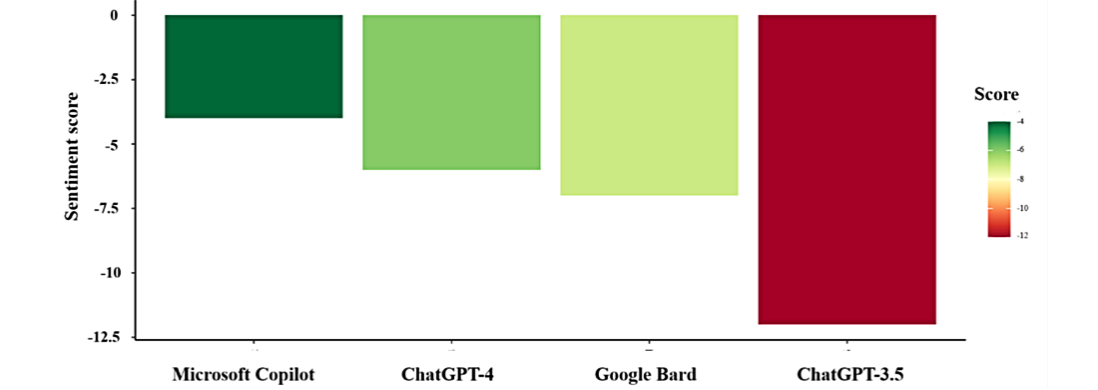
Sentiment analyses for Google Bard, Microsoft Copilot, ChatGPT-3.5, and ChatGPT-4.

In the sentiment analysis, Microsoft Copilot had the least negative score (–4), reflecting a neutral and balanced tone. ChatGPT-4 scored (–6), slightly more negative due to its detailed discussion of risks. Google Bard followed with (–7), emphasizing practical recommendations that highlighted potential challenges. ChatGPT-3.5 had the most negative score (–12), reflecting a strong focus on risks and adverse outcomes. These differences, shown in Figure 1, highlight the tonal variations among the models, with Microsoft Copilot being the most neutral and ChatGPT-3.5 the most risk-oriented.

## Discussion

### Overview of Major Study Findings

This study comprehensively assessed the knowledge, clarity, and objectivity of 4 prominent LLMs (ChatGPT-3.5, ChatGPT-4, Microsoft Copilot, and Google Bard) regarding COVID-19 impacts on pregnancy. Aligning with the stated aims, the findings revealed distinct performance variations among the LLMs. ChatGPT-4 and Microsoft Copilot demonstrated the highest levels of accuracy (97%, 32/33), followed by Google Bard (94%, 31/33) and ChatGPT-3.5 (82%, 27/33). While all models provided generally reliable information on COVID-19 and vaccination in maternal health, they varied in readability, with Microsoft Copilot being the most accessible and ChatGPT-3.5 the least comprehensible. Sentiment analysis highlighted Microsoft Copilot as the most neutral, whereas ChatGPT-3.5 displayed a risk-heavy tone. These outcomes underscored the evolving potential of LLMs to support health care professionals and public health communication during global crises while emphasizing the necessity for ongoing evaluation and model refinement to align with the latest scientific evidence.

#### The Four LLMs Differ in Terms of Knowledge, With ChatGPT-3.5 Being the Least Knowledgeable

The performance of AI models such as ChatGPT-4, Google Bard, and Microsoft Copilot proved exceptional, with only minor errors detected. Their capacity to deliver accurate and detailed information showcased a depth of knowledge that is particularly impressive when juxtaposed with the level of COVID-19-related expertise observed among obstetrics and gynecology specialists. This comparison is drawn from findings in a previous survey [21], which revealed an average knowledge score of 75.6 (SD 10.6) among healthcare professionals in the field. Notably, while 81.1% (129/159) of these specialists displayed adequate knowledge of general COVID-19 information, a much smaller percentage (19/159, 11.9%) demonstrated specific understanding related to pregnancy, childbirth, and breastfeeding in the context of the pandemic. Furthermore, only 40.3% (64/159) of respondents showed familiarity with COVID-19 vaccination guidelines for pregnant individuals. A particularly critical area of knowledge, the increased risk of preeclampsia in pregnant women infected with COVID-19, was correctly identified by only 27% (43/159) of participants. This highlights a significant gap in awareness that could impact patient care. Regarding immunization practices, about 65% (104/159) of respondents recommended COVID-19 vaccination during all trimesters, reflecting a broad acceptance of its safety and efficacy across pregnancy stages. However, preferences for specific trimesters varied: about 25% (39/159) recommended vaccination during the second trimester, 6% (9/159) in the third trimester, and a minority of 4% (6/159) in the first trimester.

These findings align with existing literature, which consistently highlights a trend where ChatGPT-3 underperformed compared to other LLMs in specific tasks or benchmarks. Studies have noted that while ChatGPT-3 demonstrated proficiency in generating coherent text and answering general questions, it often fell short in accuracy, contextual understanding, and handling of complex queries. This performance gap has been attributed to differences in training data, model architecture, and parameter tuning [22,23]. Conversely, studies have shown that more advanced LLMs, such as ChatGPT-4 and Microsoft Copilot, outperformed ChatGPT-3, with Google Bard following closely behind [24], consistent with our findings.

However, other research suggests that ChatGPT-3.5 performed comparably or even better than other LLMs in certain contexts, potentially indicating that performance may vary depending on the specific application or field of use [25].

#### The Four LLMs Differ in Terms of Recommendations of COVID-19 Vaccination During Pregnancy, With ChatGPT-3.5 Being the Least Aligned With Scientific Evidence

When comparing the information provided about COVID-19 in pregnant women and the vaccination during pregnancy, there were some notable variations, especially in alignment with scientific evidence. Concerning the risk of severe illness, Google Bard highlighted an increased risk of severe complications, including hospitalization, intensive care unit admission, and death for pregnant women. In addition, it pointed out a higher likelihood of preterm birth. Microsoft Copilot emphasized that, while pregnant women are not more likely to contract the virus, they face a greater risk of severe disease, especially if infected in the third trimester. ChatGPT-3.5 noted only a slightly higher risk of severe illness for pregnant women compared to nonpregnant individuals, including increased hospitalization and intensive care unit admission rates, while, more correctly, ChatGPT-4 stated that pregnant women with COVID-19 have a higher risk of severe illness and complications, such as preterm birth. Regarding COVID-19 vaccination during pregnancy, Google Bard strongly advocated for COVID-19 immunization during pregnancy, highlighting its safety and effectiveness in preventing serious illness and protecting both mother and baby. Microsoft Copilot stressed the importance of vaccination for those women who are pregnant, breastfeeding, or planning pregnancy, emphasizing its safety and efficacy. ChatGPT-3.5 discussed the safety of Messenger Ribonucleic Acid vaccines for pregnant individuals and suggested discussing the timing of vaccination with health care providers, while ChatGPT-4 recommended COVID-19 vaccines for pregnant individuals, noting their safety and efficacy and also advised discussing vaccination timing with health care providers. In terms of vaccine safety and side effects, Google Bard mentioned that the side effects of vaccination in pregnant women are similar to those in nonpregnant adults and are generally mild and short-lived. Microsoft Copilot, ChatGPT-3.5, and ChatGPT-4 all emphasized the safety of Messenger Ribonucleic Acid COVID-19 vaccines during pregnancy and did not report any serious adverse maternal or fetal effects. Finally, Google Bard advised pregnant women to get vaccinated as soon as possible after the first trimester, whereas ChatGPT-4 suggested postvaccination monitoring for pregnant women through programs like Centers for Disease Control and Prevention’s V-safe and emphasized the importance of consulting healthcare providers. All sources agreed on the increased risk of severe COVID-19 in pregnant women and the benefits and safety of vaccination during pregnancy. However, they differed in their emphasis on specific risks and considerations.

The comparison of LLMs in providing information about COVID-19 in pregnant women and vaccination during pregnancy underscores their potential to disseminate health information effectively but also highlights significant variability in alignment with scientific evidence. This variability raises concerns about the accuracy and reliability of AI-generated health content, particularly when subtle differences in emphasis or framing could influence public perception and decision-making. The findings illustrate how LLMs like ChatGPT, Microsoft Copilot, and Google Bard can either reinforce or undermine public health messaging, depending on their training data and underlying algorithms. These differences emphasize the need for rigorous evaluation and validation of AI-generated content in healthcare contexts, as inconsistencies could amplify the risk of misinformation, contributing to the emerging “AI-driven infodemic” [26]. Establishing robust mechanisms for quality control and promoting transparency in AI outputs is imperative to mitigate these risks and ensure that LLMs support, rather than hinder, public health objectives.

#### The Four LLMs Differ in Terms of Vocabulary, Clarity, and Readability, With ChatGPT-3.5 Being the Most Difficult to Understand

From the text-mining analysis, overall, while there was a significant overlap in key themes across all four sources, each one brought its unique focus and depth to the discussion. The unique terms in each source highlighted their different approaches to discussing the same topic, reflecting the diversity in how information can be presented and prioritized. Google Bard and Microsoft Copilot tended to have a more general approach, while ChatGPT versions provided a more detailed, medically-oriented perspective. In particular, ChatGPT-3.5, being excessively technical, is particularly difficult to understand, as also reflected by the Flesch-Kincaid grade level analysis.

The findings from our text-mining analysis align closely with a comparative study [27], which showed that ChatGPT-3.5 tended to generate overly technical content that may hinder readability.

The differences in readability and complexity across LLMs are important when considering the target audience for the information [28-30]. For a general audience, a lower Flesch-Kincaid Grade Level might be more appropriate to ensure wider comprehension. On the other hand, for professional or academic audiences, a higher grade level could be more suitable, as it allows for the conveyance of complex ideas using specialized vocabulary and detailed explanations. Each LLM's approach reflects its unique language processing capabilities and potentially the nature of the training data it was exposed to. It is of crucial importance for users to consider the context and audience when evaluating or choosing the most appropriate AI tool for generating text, with ChatGPT’s and Microsoft Copilot’s approach appearing more beneficial for specialist or academic audiences and for the general public, respectively.

#### The Four LLMs Differ in Terms of Sentiment and Objectivity, With ChatGPT-3.5 Being the Least Factual

In the sentiment analysis, the tools demonstrated varying degrees of negativity in their language when discussing COVID-19 and pregnancy-related topics. Microsoft Copilot achieved the least negative score (–4), indicating a relatively neutral or balanced tone compared to its counterparts. This suggests that Microsoft Copilot’s responses tended to minimize emotionally charged or negative language, focusing instead on providing concise, objective, and factual information. ChatGPT-4 followed with a slightly more negative score of –6, reflecting a balanced but somewhat more cautious tone. This could be attributed to ChatGPT-4’s detailed discussion of potential risks and complications, which, while comprehensive, likely included language that leaned toward highlighting challenges and considerations in the context of COVID-19 and pregnancy. Google Bard scored –7, placing it closer to ChatGPT-4 in terms of sentiment but slightly more negative. This may be indicative of its focus on delivering actionable recommendations and clinical insights, which, while practical, may also involve language emphasizing adverse effects or potential risks, thereby increasing its overall negativity score. ChatGPT-3.5 obtained the most negative score of –12, representing a significantly more cautious or risk-focused tone compared to the other models. This heightened negativity could reflect the model's tendency to elaborate extensively on risks and adverse outcomes, potentially overemphasizing negative aspects in its explanations.

These differences in sentiment scores highlight the varying tonal approaches of the AI models. Microsoft Copilot's minimal negativity suggests a focus on general, less emotionally charged language, while ChatGPT-3.5’s highest negativity score underscores its more risk-heavy narrative. ChatGPT-4 and Google Bard occupy the middle ground, balancing detailed explanations with practical recommendations, albeit with some cautionary emphasis. This variation in sentiment reflects not only the linguistic choices of the models but also their inherent design priorities, ranging from objectivity and balance to detailed elaboration and actionable advice.

### Strengths and Limitations

This study has several notable strengths. First, the use of a validated questionnaire ensures a robust and reliable assessment of LLMs' proficiency, clarity, and objectivity. The evaluation spans multiple dimensions, including knowledge, sentiment, and readability, offering a comprehensive analysis of the capabilities of ChatGPT-3.5, ChatGPT-4, Google Bard, and Microsoft Copilot. In addition, the comparison against specialist knowledge provides a meaningful benchmark, grounding the findings in real-world relevance. The inclusion of sentiment and readability analyses highlights the accessibility and emotional tone of AI-generated responses, further contextualizing their suitability for different audiences. Moreover, the study addresses a critical gap in assessing AI tools in the context of obstetrics, gynecology, and public health during a pandemic—a time when accurate information is essential. By incorporating text-mining and readability metrics, the research elucidates how LLMs can serve diverse user bases, ranging from healthcare professionals to general audiences. However, this study is not without its limitations. A key limitation is the static nature of the evaluation, as LLMs undergo continuous updates and learning processes. This dynamic evolution may render some of the current observations obsolete over time, necessitating periodic re-evaluation. Furthermore, the study focuses on a specific application of LLMs (COVID-19’s impacts in pregnancy), which may not generalize to other medical or public health domains. The zero-shot prompting approach, while simulating real-world user queries, may not fully capture the potential of these models when used with tailored or fine-tuned inputs. Finally, the study is limited by its reliance on the English language and the specific design of the validated questionnaire. Future research could explore the multilingual capabilities of LLMs and their application to other health care domains, as well as integrate expert feedback during iterative model evaluation.

### Future Directions

The present comprehensive analysis covering various aspects (knowledge, recommendations, vocabulary and readability, sentiment, and objectivity) shows similarities and dissimilarities among four major LLMs.

The knowledge appraisal highlights a gap in awareness of COVID-19 and immunization during pregnancy among specialists, underscoring the potential role of AI like ChatGPT in supplementing healthcare knowledge and decision-making. Generative AI holds great potential in health care and global public health, serving as valuable tools for professionals, providing quick access to updated information, and filling knowledge gaps.

However, some discrepancies in ChatGPT-3.5's responses also serve as a reminder of the need for continuous updating and verification of AI knowledge bases. This ensures that the information provided is aligned with the latest research and clinical guidelines. As AI systems become more integrated into health care, their ability to adapt and learn from emerging data will be crucial in ensuring they remain reliable and beneficial adjuncts in medical practice [31,32].

Future research directions should consider the inclusion of emerging AI models to evaluate their comparative utility in clinical settings. Investigating the effectiveness of these tools across various medical specialties could shed light on their strengths and limitations. Studies assessing the real-world integration of LLMs into health care workflows, focusing on usability, clinician satisfaction, and patient outcomes, are also needed. In addition, exploring the ethical and legal implications of using AI in clinical practice (such as ensuring patient privacy, safeguarding data security, and clarifying liability in decision-making) remains a crucial area of inquiry. Longitudinal studies on the influence of AI tools on medical education, professional development, health care delivery, and communication would provide valuable insights into their sustained impact.

Overall, the analysis underscores the importance of choosing the right LLM for specific needs. While ChatGPT-4 and newer versions offer more detailed and updated information, earlier versions like ChatGPT-3.5 might present challenges in terms of comprehensibility. The choice between these LLMs should be influenced by the target audience and the level of detail and technicality required in the information. For general audiences, simpler language (as seen in Microsoft Copilot) is preferable, whereas more specialized audiences might benefit from the detailed approach of ChatGPT-4. For instance, in vaccine management, ChatGPT-4 can support specialized audiences by aiding clinical decision-making, providing detailed research insights, and tailoring vaccination guidelines for pregnant women. Microsoft Copilot excels in communicating simplified information to general audiences, making it ideal for educating patients, training health workers, or addressing vaccine hesitancy. Google Bard, with its real-time search integration, is suited for disseminating up-to-date policies and driving public health campaigns with clear, actionable messaging. ChatGPT-3.5, despite its limitations, can be used for simulating scenarios or gathering feedback to refine communication strategies. These applications demonstrate how aligning LLMs’ capabilities with target audiences ensures effective vaccine management and can be potentially translated to other clinical settings and patient management contexts.

### Conclusions

This study highlighted the strengths and limitations of LLMs such as ChatGPT-3.5, ChatGPT-4, Microsoft Copilot, and Google Bard in addressing critical healthcare topics, specifically the impacts of COVID-19 on pregnancy. While the findings underscored the potential of LLMs to provide accurate, up-to-date information, they also revealed gaps in their knowledge and variability in their communication styles, sentiment, and readability. Such variability underscores the importance of tailoring AI use to specific audiences and contexts. Beyond the immediate findings, the broader implications of this research emphasize the transformative potential of LLMs in health care and public health communication. These AI tools can bridge knowledge gaps among health care professionals, support evidence-based decision-making, and combat misinformation during health crises. Their ability to deliver clear, accessible, and accurate information positions them as valuable adjuncts in both clinical settings and public health campaigns. However, the study also underscores critical ethical and operational considerations. Ensuring the reliability of AI-generated content requires continuous updates to training datasets and rigorous validation against the latest scientific evidence. The dynamic nature of AI development means that its integration into health care systems must be accompanied by robust oversight mechanisms to mitigate risks such as the dissemination of misinformation or unintentional biases. Furthermore, the deployment of LLMs in health care highlights the need for interdisciplinary collaboration among AI developers, health care professionals, and policy makers. Such partnerships can ensure that these tools are designed and utilized in ways that prioritize patient safety, data privacy, and equitable access to information. By fostering these collaborations, the integration of AI into health care can move beyond theoretical potential to practical, impactful applications. In conclusion, while LLMs like ChatGPT-4 and Microsoft Copilot demonstrate remarkable capabilities in providing health care–related information, their limitations remind us of the essential role of human oversight. The findings of this study advocate for a future where AI complements, rather than replaces, human expertise in healthcare, enhancing both the quality and accessibility of medical knowledge. This balanced approach will be key to leveraging AI's full potential while safeguarding the ethical and practical dimensions of health care delivery.

## Appendix

Multimedia Appendix 1 Overview of queries and prompts used with Google Bard, Microsoft Copilot, ChatGPT-3.5, and ChatGPT-4.

Multimedia Appendix 2 Responses provided by each large language model.

Multimedia Appendix 3 Responses generated by each large language model (Google Bard, Microsoft Copilot, ChatGPT-3.5, and ChatGPT-4) when prompted to provide recommendations on COVID-19 vaccination for pregnant women.

## Abbreviations

AI: artificial intelligence
ART: assisted reproductive technology
LLM: large language model
TDM: Term-Document Matrix
WHO: World Health Organization

## Data Availability

All data are available in Multimedia Appendices 1-3.
