# Supplementary material for: Proficiency, Clarity, and Objectivity of Large Language Models Versus Specialists’ Knowledge on COVID-19's Impacts in Pregnancy: Cross-Sectional Pilot Study
Source: JMIR Form Res. 2025 Feb 5;9:e56126. doi: 10.2196/56126 (PMC11840386; doi:10.2196/56126)
Supplement: Multimedia Appendix 1 [file formative_v9i1e56126_app1.docx]

**Overview of queries and prompts used with Google Bard, Microsoft Copilot, ChatGPT-3.5, and ChatGPT-4.**

Please state whether these statements are false or true or complete the statement where appropriate.

1. COVID-19 is transmitted through respiratory droplets.
2. Hand washing protects against COVID-19.
3. Face masks protect against COVID-19.
4. Incubation period for COVID-19 is 2-14 days.
5. To reduce the risk of infection, people with COVID-19 should be isolated and treated.
6. Pregnant women are at a higher risk of developing COVID-19 than non-pregnant women.
7. Pregnant women with COVID-19 have more complications compared to non-pregnant women of the same age.
8. Pregnant women with COVID-19 should take additional precautions to protect themselves from COVID-19, more than non-pregnant women of the same age.
9. Pregnant and postpartum women with COVID-19 can take NSAIDs and acetaminophen.
10. COVID-19 positive pregnant patients are more likely to have preterm labour, compared to pregnant women who do not have COVID-19.
11. Compared with pregnant women of similar age, pregnant women with COVID-19 are more prone to hospitalizations, intensive care hospitalizations, and artificial respiration.
12. Pregnant women with COVID-19 have a higher risk of developing preeclampsia compared to pregnant women who do not have COVID-19.
13. Pregnant women with COVID-19 have an increased risk of miscarriage.
14. Pregnant women with COVID-19 have an increased risk of thromboembolic events, compared to non-pregnant women and COVID-19 patients.
15. Vertical transmission appears to be uncommon.
16. Pregnant women with COVID-19 can pass the virus onto the foetus or baby intrauterine or during delivery.
17. Caesarean section is indicated for the delivery of all pregnant COVID-19 patients.
18. A COVID-19 infected mother can transmit the virus to the baby through contact or respiratory droplets during breastfeeding.
19. COVID-19 can be transmitted through breast milk.
20. How many types of COVID-19 vaccines have been approved for use by the FDA?
21. The Pfizer and Moderna COVID-19 vaccines are messenger RNA vaccines.
22. The AstraZeneca vaccine is a viral vector vaccine.
23. Based on results of clinical trials, Pfizer and Moderna vaccines are effective in preventing symptomatic COVID-19 disease in individuals who received two doses with no evidence of previous infection at …
24. Thrombosis with thrombocytopenia syndrome was identified in patients who received …
25. Clinical trials have confirmed that COVID-19 vaccines adversely affect fertility and foetal development.
26. To date, the v-safe registry data on the reactogenicity profile and side-effects in pregnant women have not indicated any safety concerns.
27. The rate and range of side-effects from the vaccines are similar in pregnant and non-pregnant women.
28. The effectiveness of the vaccines can vary depending on the length of time that has elapsed since the vaccine and the viral strain.
29. Based on the information accumulated so far, a booster dose increases the effectiveness of the vaccine.
30. It is permissible to give COVID-19 vaccine simultaneously with vaccines recommended to pregnant women, such as vaccine against pertussis or influenza, or at any time before or after these vaccines.
31. It is best not to give the vaccine to woman who are planning pregnancy because the vaccine affects fertility.
32. A woman who received the first dose of the vaccine and became pregnant - it is recommended that she complete the second and third doses of the vaccine according to the accepted schedule.
33. Coronavirus vaccine should not be given to women who are breastfeeding.

Would you recommend COVID-19 vaccine to all pregnant women (without contraindication)?

Would you recommend the vaccine only to pregnant women who are at high risk of contracting the virus?

Would you recommend COVID-19 vaccine to all women (without contraindication) of reproductive age who are not pregnant?

Would you recommend vaccination of all women (without contraindication) planning to undergo assisted reproduction?

Would you recommend an interval between vaccination and pregnancy?
