# Supplementary material for: Proficiency, Clarity, and Objectivity of Large Language Models Versus Specialists’ Knowledge on COVID-19's Impacts in Pregnancy: Cross-Sectional Pilot Study
Source: JMIR Form Res. 2025 Feb 5;9:e56126. doi: 10.2196/56126 (PMC11840386; doi:10.2196/56126)
Supplement: Multimedia Appendix 2 [file formative_v9i1e56126_app2.docx]

**Responses provided by each Large Language Model.**

**Google Bard**

1. COVID-19 is transmitted through respiratory droplets. **True**
2. Hand washing protects against COVID-19. **True**
3. Face masks protect against COVID-19. **True**
4. Incubation period for COVID-19 is 2-14 days. **True**
5. To reduce the risk of infection, people with COVID-19 should be isolated and treated. **True**
6. Pregnant women are at a higher risk of developing COVID-19 than non-pregnant women. **False**
7. Pregnant women with COVID-19 have more complications compared to non-pregnant women of the same age. **True**
8. Pregnant women with COVID-19 should take additional precautions to protect themselves from COVID-19, more than non-pregnant women of the same age. **True**
9. Pregnant and postpartum women with COVID-19 can take NSAIDs and acetaminophen. **True**
10. COVID-19 positive pregnant patients are more likely to have preterm labour, compared to pregnant women who do not have COVID-19. **True**
11. Compared with pregnant women of similar age, pregnant women with COVID-19 are more prone to hospitalizations, intensive care hospitalizations, and artificial respiration. **True**
12. Pregnant women with COVID-19 have a higher risk of developing preeclampsia compared to pregnant women who do not have COVID-19. **True**
13. Pregnant women with COVID-19 have an increased risk of miscarriage. **False**
14. Pregnant women with COVID-19 have an increased risk of thromboembolic events, compared to non-pregnant women and COVID-19 patients. **True**
15. Vertical transmission appears to be uncommon. **True**
16. Pregnant women with COVID-19 can pass the virus onto the foetus or baby intrauterine or during delivery. **False**
17. Caesarean section is indicated for the delivery of all pregnant COVID-19 patients. **False**
18. A COVID-19 infected mother can transmit the virus to the baby through contact or respiratory droplets during breastfeeding. **False**
19. COVID-19 can be transmitted through breast milk. **False**
20. How many types of COVID-19 vaccines have been approved for use by the FDA? **3**
21. The Pfizer and Moderna COVID-19 vaccines are messenger RNA vaccines. **True**
22. The AstraZeneca vaccine is a viral vector vaccine. **True**
23. Based on results of clinical trials, Pfizer and Moderna vaccines are effective in preventing symptomatic COVID-19 disease in individuals who received two doses with no evidence of previous infection at … **95%**
24. Thrombosis with thrombocytopenia syndrome was identified in patients who received … **Johnson & Johnson (Janssen)**
25. Clinical trials have confirmed that COVID-19 vaccines adversely affect fertility and foetal development. **False**
26. To date, the v-safe registry data on the reactogenicity profile and side-effects in pregnant women have not indicated any safety concerns. **True**
27. The rate and range of side-effects from the vaccines are similar in pregnant and non-pregnant women. **True**
28. The effectiveness of the vaccines can vary depending on the length of time that has elapsed since the vaccine and the viral strain. **True**
29. Based on the information accumulated so far, a booster dose increases the effectiveness of the vaccine. **True**
30. It is permissible to give COVID-19 vaccine simultaneously with vaccines recommended to pregnant women, such as vaccine against pertussis or influenza, or at any time before or after these vaccines. **True**
31. It is best not to give the vaccine to woman who are planning pregnancy because the vaccine affects fertility. **False**
32. A woman who received the first dose of the vaccine and became pregnant - it is recommended that she complete the second and third doses of the vaccine according to the accepted schedule. **True**
33. Coronavirus vaccine should not be given to women who are breastfeeding. **False**

**Microsoft Copilot**

1. COVID-19 is transmitted through respiratory droplets. **True**
2. Hand washing protects against COVID-19. **True**
3. Face masks protect against COVID-19. **True**
4. Incubation period for COVID-19 is 2-14 days. **True**
5. To reduce the risk of infection, people with COVID-19 should be isolated and treated. **True**
6. Pregnant women are at a higher risk of developing COVID-19 than non-pregnant women. **False**
7. Pregnant women with COVID-19 have more complications compared to non-pregnant women of the same age. **False**
8. Pregnant women with COVID-19 should take additional precautions to protect themselves from COVID-19, more than non-pregnant women of the same age. **True**
9. Pregnant and postpartum women with COVID-19 can take NSAIDs and acetaminophen. **True**
10. COVID-19 positive pregnant patients are more likely to have preterm labour, compared to pregnant women who do not have COVID-19. **True**
11. Compared with pregnant women of similar age, pregnant women with COVID-19 are more prone to hospitalizations, intensive care hospitalizations, and artificial respiration. **True**
12. Pregnant women with COVID-19 have a higher risk of developing preeclampsia compared to pregnant women who do not have COVID-19. **True**
13. Pregnant women with COVID-19 have an increased risk of miscarriage. **False**
14. Pregnant women with COVID-19 have an increased risk of thromboembolic events, compared to non-pregnant women and COVID-19 patients. **True**
15. Vertical transmission appears to be uncommon. **True**
16. Pregnant women with COVID-19 can pass the virus onto the foetus or baby intrauterine or during delivery. **True**
17. Caesarean section is indicated for the delivery of all pregnant COVID-19 patients. **False**
18. A COVID-19 infected mother can transmit the virus to the baby through contact or respiratory droplets during breastfeeding. **True**
19. COVID-19 can be transmitted through breast milk. **False**
20. How many types of COVID-19 vaccines have been approved for use by the FDA? **3**
21. The Pfizer and Moderna COVID-19 vaccines are messenger RNA vaccines. **True**
22. The AstraZeneca vaccine is a viral vector vaccine. **True**
23. Based on results of clinical trials, Pfizer and Moderna vaccines are effective in preventing symptomatic COVID-19 disease in individuals who received two doses with no evidence of previous infection at … **95%**
24. Thrombosis with thrombocytopenia syndrome was identified in patients who received … **Johnson & Johnson (Janssen)**
25. Clinical trials have confirmed that COVID-19 vaccines adversely affect fertility and foetal development. **False**
26. To date, the v-safe registry data on the reactogenicity profile and side-effects in pregnant women have not indicated any safety concerns. **True**
27. The rate and range of side-effects from the vaccines are similar in pregnant and non-pregnant women. **True**
28. The effectiveness of the vaccines can vary depending on the length of time that has elapsed since the vaccine and the viral strain. **True**
29. Based on the information accumulated so far, a booster dose increases the effectiveness of the vaccine. **True**
30. It is permissible to give COVID-19 vaccine simultaneously with vaccines recommended to pregnant women, such as vaccine against pertussis or influenza, or at any time before or after these vaccines. **True**
31. It is best not to give the vaccine to woman who are planning pregnancy because the vaccine affects fertility. **False**
32. A woman who received the first dose of the vaccine and became pregnant - it is recommended that she complete the second and third doses of the vaccine according to the accepted schedule. **True**
33. Coronavirus vaccine should not be given to women who are breastfeeding. **False**

**ChatGPT-3.5**

1. COVID-19 is transmitted through respiratory droplets. **True**
2. Hand washing protects against COVID-19. **True**
3. Face masks protect against COVID-19. **True**
4. Incubation period for COVID-19 is 2-14 days. **True**
5. To reduce the risk of infection, people with COVID-19 should be isolated and treated. **True**
6. Pregnant women are at a higher risk of developing COVID-19 than non-pregnant women. **True**
7. Pregnant women with COVID-19 have more complications compared to non-pregnant women of the same age. **False**
8. Pregnant women with COVID-19 should take additional precautions to protect themselves from COVID-19, more than non-pregnant women of the same age. **True**
9. Pregnant and postpartum women with COVID-19 can take NSAIDs and acetaminophen. **False**
10. COVID-19 positive pregnant patients are more likely to have preterm labour, compared to pregnant women who do not have COVID-19. **True**
11. Compared with pregnant women of similar age, pregnant women with COVID-19 are more prone to hospitalizations, intensive care hospitalizations, and artificial respiration. **True**
12. Pregnant women with COVID-19 have a higher risk of developing preeclampsia compared to pregnant women who do not have COVID-19. **True**
13. Pregnant women with COVID-19 have an increased risk of miscarriage. **True**
14. Pregnant women with COVID-19 have an increased risk of thromboembolic events, compared to non-pregnant women and COVID-19 patients. **True**
15. Vertical transmission appears to be uncommon. **True**
16. Pregnant women with COVID-19 can pass the virus onto the foetus or baby intrauterine or during delivery. **False**
17. Caesarean section is indicated for the delivery of all pregnant COVID-19 patients. **False**
18. A COVID-19 infected mother can transmit the virus to the baby through contact or respiratory droplets during breastfeeding. **True**
19. COVID-19 can be transmitted through breast milk. **True**
20. How many types of COVID-19 vaccines have been approved for use by the FDA? **3**
21. The Pfizer and Moderna COVID-19 vaccines are messenger RNA vaccines. **True**
22. The AstraZeneca vaccine is a viral vector vaccine. **True**
23. Based on results of clinical trials, Pfizer and Moderna vaccines are effective in preventing symptomatic COVID-19 disease in individuals who received two doses with no evidence of previous infection at … **95%**
24. Thrombosis with thrombocytopenia syndrome was identified in patients who received … **Johnson & Johnson (Janssen)**
25. Clinical trials have confirmed that COVID-19 vaccines adversely affect fertility and foetal development. **False**
26. To date, the v-safe registry data on the reactogenicity profile and side-effects in pregnant women have not indicated any safety concerns. **True**
27. The rate and range of side-effects from the vaccines are similar in pregnant and non-pregnant women. **True**
28. The effectiveness of the vaccines can vary depending on the length of time that has elapsed since the vaccine and the viral strain. **True**
29. Based on the information accumulated so far, a booster dose increases the effectiveness of the vaccine. **True**
30. It is permissible to give COVID-19 vaccine simultaneously with vaccines recommended to pregnant women, such as vaccine against pertussis or influenza, or at any time before or after these vaccines. **True**
31. It is best not to give the vaccine to woman who are planning pregnancy because the vaccine affects fertility. **False**
32. A woman who received the first dose of the vaccine and became pregnant - it is recommended that she complete the second and third doses of the vaccine according to the accepted schedule. **True**
33. Coronavirus vaccine should not be given to women who are breastfeeding. **False**

**ChatGPT-4**

1. COVID-19 is transmitted through respiratory droplets. **True**
2. Hand washing protects against COVID-19. **True**
3. Face masks protect against COVID-19. **True**
4. Incubation period for COVID-19 is 2-14 days. **True**
5. To reduce the risk of infection, people with COVID-19 should be isolated and treated. **True**
6. Pregnant women are at a higher risk of developing COVID-19 than non-pregnant women. **False**
7. Pregnant women with COVID-19 have more complications compared to non-pregnant women of the same age. **True**
8. Pregnant women with COVID-19 should take additional precautions to protect themselves from COVID-19, more than non-pregnant women of the same age. **True**
9. Pregnant and postpartum women with COVID-19 can take NSAIDs and acetaminophen. **True**
10. COVID-19 positive pregnant patients are more likely to have preterm labour, compared to pregnant women who do not have COVID-19. **True**
11. Compared with pregnant women of similar age, pregnant women with COVID-19 are more prone to hospitalizations, intensive care hospitalizations, and artificial respiration. **True**
12. Pregnant women with COVID-19 have a higher risk of developing preeclampsia compared to pregnant women who do not have COVID-19. **True**
13. Pregnant women with COVID-19 have an increased risk of miscarriage. **True**
14. Pregnant women with COVID-19 have an increased risk of thromboembolic events, compared to non-pregnant women and COVID-19 patients. **True**
15. Vertical transmission appears to be uncommon. **True**
16. Pregnant women with COVID-19 can pass the virus onto the foetus or baby intrauterine or during delivery. **True**
17. Caesarean section is indicated for the delivery of all pregnant COVID-19 patients. **False**
18. A COVID-19 infected mother can transmit the virus to the baby through contact or respiratory droplets during breastfeeding. **True**
19. COVID-19 can be transmitted through breast milk. **False**
20. How many types of COVID-19 vaccines have been approved for use by the FDA? **3**
21. The Pfizer and Moderna COVID-19 vaccines are messenger RNA vaccines. **True**
22. The AstraZeneca vaccine is a viral vector vaccine. **True**
23. Based on results of clinical trials, Pfizer and Moderna vaccines are effective in preventing symptomatic COVID-19 disease in individuals who received two doses with no evidence of previous infection at … **95%**
24. Thrombosis with thrombocytopenia syndrome was identified in patients who received … **Johnson & Johnson (Janssen)**
25. Clinical trials have confirmed that COVID-19 vaccines adversely affect fertility and foetal development. **False**
26. To date, the v-safe registry data on the reactogenicity profile and side-effects in pregnant women have not indicated any safety concerns. **True**
27. The rate and range of side-effects from the vaccines are similar in pregnant and non-pregnant women. **True**
28. The effectiveness of the vaccines can vary depending on the length of time that has elapsed since the vaccine and the viral strain. **True**
29. Based on the information accumulated so far, a booster dose increases the effectiveness of the vaccine. **True**
30. It is permissible to give COVID-19 vaccine simultaneously with vaccines recommended to pregnant women, such as vaccine against pertussis or influenza, or at any time before or after these vaccines. **True**
31. It is best not to give the vaccine to woman who are planning pregnancy because the vaccine affects fertility. **False**
32. A woman who received the first dose of the vaccine and became pregnant - it is recommended that she complete the second and third doses of the vaccine according to the accepted schedule. **True**
33. Coronavirus vaccine should not be given to women who are breastfeeding. **False**
