# Supplementary material for: Proficiency, Clarity, and Objectivity of Large Language Models Versus Specialists’ Knowledge on COVID-19's Impacts in Pregnancy: Cross-Sectional Pilot Study
Source: JMIR Form Res. 2025 Feb 5;9:e56126. doi: 10.2196/56126 (PMC11840386; doi:10.2196/56126)
Supplement: Multimedia Appendix 3 [file formative_v9i1e56126_app3.docx]

**Responses generated by each large language model (Google Bard, Microsoft Copilot, ChatGPT-3.5, and ChatGPT-4) when prompted to provide recommendations on COVID-19 vaccination for pregnant women.**

**Google Bard**

While COVID-19 generally affects pregnant women similarly to non-pregnant adults, there are some unique considerations and risks associated with the virus during pregnancy. Pregnant women are at an increased risk of developing severe complications from COVID-19, including hospitalization, intensive care unit (ICU) admission, and even death. They are also more likely to experience preterm birth or other pregnancy complications. Pregnant women with certain underlying medical conditions, such as obesity, diabetes, asthma, or chronic hypertension, are at an even higher risk of severe COVID-19 complications. In addition, older pregnant women (≥35 years old) are also more vulnerable to severe illness. COVID-19 vaccination is highly effective at preventing serious illness, hospitalization, and death from COVID-19 in pregnant women. It is also safe and effective for pregnant women of all ages and medical conditions. Vaccination during pregnancy can also help to protect the baby from COVID-19, as antibodies from the mother can pass through the placenta and provide some protection for the newborn. The Centers for Disease Control and Prevention (CDC) strongly recommends COVID-19 vaccination for all pregnant women, regardless of their medical history or whether they have been exposed to COVID-19. The CDC also recommends that pregnant women get vaccinated as soon as possible after their first trimester, if possible. The side effects of COVID-19 vaccination in pregnant women are similar to those experienced by non-pregnant adults. The most common side effects are pain, redness, and swelling at the injection site, as well as fatigue, headache, muscle aches, and fever. These side effects are usually mild and short-lived. Numerous studies have shown that COVID-19 vaccination is safe and effective for pregnant women. No serious safety concerns have been identified in pregnant women or their babies who have received the vaccine. COVID-19 vaccination is the best way to protect pregnant women and their babies from COVID-19. It is safe and effective, and it can help to prevent serious illness, hospitalization, and death. Pregnant women should talk to their doctor about getting vaccinated as soon as possible.

**Microsoft Copilot**

Pregnant women do not seem to be at higher risk of getting SARS-CoV-2, the virus that causes COVID-19. However, they are at greater risk of severe disease than their non-pregnant peers if they contract the virus. This risk is particularly increased if they contract the infection in the third trimester of pregnancy. Severe COVID-19 during pregnancy can raise the risk of pregnancy problems, such as preterm birth. COVID-19 vaccination is recommended for people who are pregnant, breastfeeding, trying to get pregnant now, or might become pregnant in the future. Vaccination during pregnancy is safe and effective. It can help protect both the mother and the baby from serious illness from COVID-19. There is no evidence of adverse maternal or fetal effects from vaccinating pregnant individuals with the COVID-19 vaccine. It’s safe to get vaccinated at any point during your pregnancy. Please consult with a healthcare provider for personalized advice. This information is based on the data available as of 2023 and may have changed. Stay safe!

**ChatGPT-3.5**

COVID-19, caused by the SARS-CoV-2 virus, can affect pregnant women differently than the general population due to the physiological changes that occur during pregnancy. Here are some key points to consider regarding COVID-19 in pregnant women. Pregnant women may be at a slightly higher risk of experiencing severe illness if they contract COVID-19 compared to non-pregnant individuals of the same age. This includes a higher likelihood of hospitalization, intensive care unit (ICU) admission, and the need for mechanical ventilation. There is some evidence to suggest that COVID-19 may increase the risk of preterm birth (delivering the baby before 37 weeks of gestation) and other adverse pregnancy outcomes. Pregnant women with COVID-19 are more likely to experience symptoms such as shortness of breath, chest pain, and pneumonia. In severe cases, it can lead to acute respiratory distress syndrome (ARDS). While rare, there have been isolated cases of vertical transmission, where the virus is passed from the mother to the baby during pregnancy or childbirth. However, the risk of vertical transmission is considered low. Most babies born to mothers with COVID-19 do not have the virus at birth. There have been reports of preterm birth and lower birth weight in some cases, but it's challenging to attribute these outcomes solely to COVID-19. Vaccination against COVID-19 during pregnancy has been a topic of significant research and discussion. As of my last knowledge update in January 2022, here are some important points to consider regarding COVID-19 vaccination during pregnancy. The safety of COVID-19 vaccines during pregnancy has been a subject of extensive study. Research indicates that mRNA vaccines, such as Pfizer-BioNTech and Moderna, are generally safe for pregnant individuals. These vaccines do not contain live virus and cannot alter the recipient's DNA. COVID-19 vaccination during pregnancy offers potential benefits. It can reduce the risk of severe illness in pregnant individuals and may provide passive immunity to the newborn, as antibodies can be transferred from the mother to the baby. Pregnant individuals are encouraged to discuss COVID-19 vaccination with their healthcare providers. The timing of vaccination may vary based on individual circumstances and trimesters. Many health authorities recommend vaccination during any trimester of pregnancy. Pregnant individuals should weigh the risks and benefits of vaccination based on their individual health, the prevalence of COVID-19 in their community, and any potential exposure risks. It's essential for pregnant individuals to have open and informed discussions with their healthcare providers to make the best decision for their specific situation. Please note that recommendations and guidelines regarding COVID-19 and pregnancy may have evolved since my last update in January 2022. I strongly recommend consulting the latest information from reputable health organizations, such as the Centers for Disease Control and Prevention (CDC) or the World Health Organization (WHO), for the most current guidance on COVID-19 vaccination during pregnancy.

**ChatGPT-4**

COVID-19, caused by the SARS-CoV-2 virus, has been a significant concern for pregnant women, as they are at a higher risk of severe illness compared to non-pregnant individuals. Here are some key points regarding COVID-19 in pregnant women and the vaccination during pregnancy. Pregnant women with COVID-19 have a higher risk of severe illness and pregnancy complications, such as preterm birth. The symptoms of COVID-19 in pregnant women are similar to those in non-pregnant individuals, including fever, cough, shortness of breath, and fatigue. There's no definitive evidence suggesting that COVID-19 infection can directly harm the fetus. However, severe maternal illness can impact fetal and neonatal outcomes. COVID-19 vaccines are recommended for pregnant individuals. The vaccines have been shown to be effective and safe during pregnancy. Studies indicate that mRNA COVID-19 vaccines (like Pfizer-BioNTech and Moderna) are safe for pregnant women. They don't contain live virus and cannot cause COVID-19 in either the pregnant individual or the fetus. Pregnant women can receive the COVID-19 vaccine at any stage of pregnancy. Timing should be discussed with healthcare providers for individual circumstances. Apart from vaccination, pregnant women are advised to follow the same precautions as the general population, such as wearing masks, practicing hand hygiene, and social distancing. After vaccination, pregnant women are encouraged to enroll in vaccine safety monitoring programs, like the CDC's V-safe, to help gather more data about the vaccine's safety during pregnancy. It's crucial for pregnant women to consult with their healthcare providers to make informed decisions about COVID-19 vaccination and the management of their health during pregnancy. COVID-19 vaccines are considered safe for individuals who are breastfeeding. There is no need to avoid initiation or discontinue breastfeeding if vaccinated. This information is based on guidelines and research available as of April 2023, and it's always important to consult healthcare professionals for personalized advice and the latest updates.
